# Role of MicroRNAs in Islet Beta-Cell Compensation and Failure during Diabetes

**DOI:** 10.1155/2014/618652

**Published:** 2014-03-05

**Authors:** Valérie Plaisance, Gérard Waeber, Romano Regazzi, Amar Abderrahmani

**Affiliations:** ^1^Lille 2 University, European Genomic Institute for Diabetes (EGID), FR 3508, UMR-8199 Lille, France; ^2^Service of Internal Medicine, Hospital-University of Lausanne (CHUV), 1011 Lausanne, Switzerland; ^3^Department of Fundamental Neurosciences, University of Lausanne, 1005 Lausanne, Switzerland

## Abstract

Pancreatic beta-cell function and mass are markedly adaptive to compensate for the changes in insulin requirement observed during several situations such as pregnancy, obesity, glucocorticoids excess, or administration. This requires a beta-cell compensation which is achieved through a gain of beta-cell mass and function. Elucidating the physiological mechanisms that promote functional beta-cell mass expansion and that protect cells against death, is a key therapeutic target for diabetes. In this respect, several recent studies have emphasized the instrumental role of microRNAs in the control of beta-cell function. MicroRNAs are negative regulators of gene expression, and are pivotal for the control of beta-cell proliferation, function, and survival. On the one hand, changes in specific microRNA levels have been associated with beta-cell compensation and are triggered by hormones or bioactive peptides that promote beta-cell survival and function. Conversely, modifications in the expression of other specific microRNAs contribute to beta-cell dysfunction and death elicited by diabetogenic factors including, cytokines, chronic hyperlipidemia, hyperglycemia, and oxidized LDL. This review underlines the importance of targeting the microRNA network for future innovative therapies aiming at preventing the beta-cell decline in diabetes.

## 1. Introduction

The concentration of glucose in the blood is tightly monitored by the pancreatic islet beta-cell production of insulin. The main function of insulin is to reduce blood glucose levels by triggering the uptake and the storage of this carbohydrate by the cells of the body. The quantity of insulin released by beta-cells varies according not only to secretagogues such as glucose but also as a function of the insulin demand from target tissues. A feedback loop also exists between insulin sensitivity and insulin secretion, such that changes in sensitivity of peripheral tissues are balanced by corresponding increases in secretion, insuring preservation of euglycemia [1, 2]. A rise in the insulin demand occurs during normal body growth (from birth to early childhood periods), as a consequence of an increase in body weight and during pregnancy. To meet the requirement of insulin, beta-cells adapt both their mass and function to release sufficient insulin to maintain blood glucose homeostasis [1, 2]. Evidence for this compensatory process has been consistently provided by rodent models of obesity and diabetes and notably by the emerging availability of human pancreas necropsies [2, 3]. Indeed, beta-cell mass and function in pancreases of nondiabetic or prediabetic obese individuals is larger than in lean normoglycemic subjects [3–5]. In obesity beta-cell mass increases by 30–40% whereas insulin secretory output augments by 100% [6]. Conversely, diminished beta-cells mass and function contribute to the decrease in plasma insulin level in individuals with diabetes. Postmortem histology further a 20–65% decrement in beta-cell mass in islets from obese individuals with type 2 diabetes (T2D) when compared to BMI-matched nondiabetic subjects [3–5, 7–9]. This adaptive capacity of human islets to obesity has been confirmed in experimental murine models [10, 11]. In one study, human islets were grafted in an immunodeficient mouse strain sensitive to high fat-diet (HFD-)induced obesity [10]. This mice model is used for longitudinal studies of islets exposed to an obesogenic environment [10]. Enlarged volume of human beta-cells was observed in xenotransplanted mice fed with HFD for 12 weeks [10]. However, despite the gain of beta-cell mass and the increase in insulin expression, these mice displayed hyperglycemia. This study confirms the requirement for an appropriate number of functional beta-cells to circumvent insulin resistance [10]. Therefore, insulin deficiency in T2D may in part result from an insufficient number of functional beta-cells under conditions such as ageing, weight gain, or metabolic alterations [7, 12, 13].

Despite intensive research, current treatments of T2D do not prevent the appearance of long-term complications and, over time, can also become inefficient to insure appropriate glycemic control. This inefficacy may result from the fact that available strategies do not permit to protect beta-cells against their inescapable decline. The existing therapies with exogenous insulin or hypoglycemic agents for type 1 diabetes (T1D) are also unsatisfactory, since they do not offer a cure and are mostly insufficient for preventing the secondary complications associated with diabetes [14]. Transplantation of a sufficient number of pancreatic beta-cells can normalize blood glucose levels and may prevent the complications of diabetes [15]. However, immunosuppressive therapy is a current obstacle in transplantation and beta-cells from cadaveric donors are in such a short supply that transplants can be provided only to a limited number of patients. Regeneration of the functional beta-cell mass in patients could potentially represent an alternative to transplantation. In view of the inefficacy of the current treatments and the increasing global prevalence of diabetes [16], it is urgent to intensify efforts for developing new therapeutic strategies for both T1D and T2D. In this regard, it is tempting to postulate that strategies aiming at improving beta-cell function and mass plasticity as well as beta-cell survival under proapoptotic conditions could be of major interest for designing innovative therapeutics to prevent beta-cell decline and restore their functional adaptive ability in diabetes.

Adaptive capacity of beta-cell mass and function depends on the activity of transcriptional and translational regulators, which tightly and timely modulate the expression of genes in response to environmental cues. The noncoding microRNAs (miRNAs) are extremely important to accomplish this task [17]. MiRNAs act as translational repressors that bind to the 3′UTR of target mRNAs, leading to translational inhibition and/or messenger degradation [18, 19]. Each miRNA can have hundreds of targets, thereby triggering pleiotropic effects in beta-cells. This review provides insights into the pivotal role of miRNAs in beta-cell adaptation and failure during diabetes [20–26].

## 2. miRNAs Required for Beta-Cell Specification Fate

The regulation of the beta-cell mass in adult life results from the dynamic balance between proliferation, neogenesis, and apoptosis. The mechanisms underlying the control of these phenomena are participating also to normal pancreas development, and thereby can help in understanding the compensatory mechanisms elicited in response to environmental cues and metabolic changes [27, 28]. The pancreas derives from a pool of endodermal cells. At the initial stage, the proliferation of the progenitor cells is stimulated by growth factors and other signalling molecules produced by the surrounding mesenchyme. This process is governed by a sequential cascade including the appearance of neurogenin3 (Neurog3) [29]. The number of Neurog3 expressing cells increases and peaks at embryonic day e15.5, after which the level of this transcription factor gradually declines [29]. Neurog3 is undetectable in insulin- and glucagon-producing cells, suggesting that it is not necessary for postnatal islet function [29]. In fact, transient expression of Neurog3 is critical for temporarily allowing the lineage-committed transcription factors required for the differentiation of the endocrine progenitor cells, which will give rise to the endocrine cell subtypes within the islets [29–31]. Ablation of Neurog3 prevents the generation of all pancreatic endocrine cells in mice. Evidence for a role of miRNAs in the control of Neurog3 during pancreas development has been provided by a mice model in which the pancreatic expression of the large majority of the RNAs has been abolished. miRNAs are usually generated by RNA polymerase II. This enzyme initially yields pre-miRNA molecules containing a hairpin loop, which undergoes sequential processing including cytosolic excision of the hairpin by the ribonuclease (RNase) type III Dicer1 [32]. In mammals, the loss of the RNase III domain of Dicer1 blocks the formation of most miRNAs [32]. The islet-specific *Dicer1 *knockout mice generated using the *Pdx1*-Cre transgene survive until birth but fail to grow and die by P3 [20]. The pancreas of Dicer1-null mice displays an almost absolute loss of insulin-producing cells and there is a marked decrease in other cell types at e18.5 [20]. The defect of endocrine cells observed in the *Dicer1 *knockout mice is associated with an increase in Hes1 level and a reduction in the formation of endocrine progenitor cells expressing Neurog3 [20]. Besides the induction of Notch signaling by Hes1, a possible synergistic mechanism accounting for Neurog3 inhibition during pancreas development could be a direct control by miRNAs. Demonstration of this hypothesis has been attempted in a model for pancreatic regeneration [33]. Regeneration of beta-cells following a 50 or 70% pancreatectomy is not associated with induction of Neurog3 protein in progenitor cells despite the presence of the transcript. This result prompted the authors to propose a posttranslational control of Neurog3 expression mediated by miRNAs [33, 34]. Results from global miRNA profiling in regenerating pancreas after partial pancreatectomy have highlighted upregulation of 4 miRNAs including miR-15a, miR-15b, miR-16, and miR-195 (Table 1) [33]. All the four miRNAs are predicted to target the Neurog3 mRNA, suggesting that they could contribute to the posttranslational regulation of the transcription factor [33]. Whether these miRNAs individually contribute to pancreas development has not yet been investigated.

## 3. miRNAs Are Required for Proliferation of Progenitor Cells and Mature Beta-Cells

When progenitor cells start expressing insulin they stop dividing. However, the beta-cell mass continues to expand during fetal and postnatal growth [28, 35–37]. A process that could account for beta-cell mass expansion in rodent is replication. In normal rats, the beta-cell population approximately doubles each day starting from the 16th day after conception [36]. After birth the beta-cell population still grows but during adult life at a much slower pace [37–39]. A role for miRNAs in the control of differentiated beta-cells has been highlighted by the generation of a mice model with beta-cell specific ablation of Dicer1. Disruption of the enzyme using the rat insulin promoter 2 (RIP-)Cre transgene leads to alteration in islet morphology, reduction in beta-cell number, and impairment in glucose-induced insulin secretion [40, 41]. Marked perturbations in beta-cell expansion and mass have been reported in knockout mice for individual miRNAs. The first one for which a major role in pancreatic development has been demonstrated is miR-375 (Table 1) [22, 42]. This miRNA is highly enriched in human and mice beta-cells [22]. The importance of miR-375 in pancreatic endocrine cell development has emerged from studies in zebrafish embryos [43]. Injection of anti-miR-375 morpholinos into one- or two-stage embryos resulted in disruption of the islet cell phenotype [43]. The miR-375 KO mice have been instrumental for unveiling a role for this miRNA in beta-cell expansion besides its involvement in the control of glucose-induced insulin secretion [42]. A 30–40% decrease in beta-cell mass has been measured within islets from these mice and, strikingly, a 1.7-fold increase in alpha-cells [42]. The combined hyperglucagonemia and hypoinsulinemia in miR-375 KO animals led them to develop hyperglycemia [42]. Other miRNAs such as miR-7a have been shown to potentially contribute to beta-cell expansion during pancreatic organogenesis. miR-7a belongs to the evolutionarily conserved miR-7a/b family and is abundant in beta-cells of rodent and human islets [44]. Inhibition of miR-7a activates the mammalian target of rapamycin (mTOR, a.k.a FRAP, RAFT, or RAPT) pathway in the mouse MIN6 insulin-producing cells and in primary mouse islets [45]. Activation of this evolutionarily conserved serine/threonine protein kinase promotes beta-cell replication and expansion of the beta-cell mass [46, 47]. The mTOR pathway can be divided into two biochemically and functionally distinct multicomponent complexes termed mTOR complex 1 (mTORC1) and mTOR complex 2 (mTORC2) [48]. The two complexes are pivotal for the control of beta-cell mass although their downstream targets are distinct [48]. Disruption of miR-7a leads to upregulation of the downstream targets of mTORC1, p70S6 K, eukaryotic translation initiation factor 4E (eIF4E) and two MAPK-interacting kinases that phosphorylate eIF4E, as well as one of the essential TORC2 components, Mapkap1 [48]. Consequently, activation of the mTOR pathway caused by the suppression of miR-7a results in increased proliferation of beta-cells. So far, independent studies have shown that human beta-cells can proliferate within islets or under *in vitro* condition but the rate is extremely low [3, 49–53]. It is noteworthy that the authors have observed a nearly 30-fold increase in human beta-cell proliferation upon the reduction of miR-7a level [48].

## 4. miRNAs Regulating Nutrient-Induced Insulin Secretion and Insulin Production

The function of mature beta-cells is to release appropriate amounts of insulin in response to its main physiological stimulus, glucose, and to other secretagogues, including the incretin hormone glucagon like peptide-1 (GLP-1) and its mimetics [54]. In fact, GLP-1 potentiates glucose-induced insulin secretion by interacting with the GLP-1 receptor [54]. In human islet beta-cells, efficient incretin-stimulated insulin secretion relies on the recruitment of a highly coordinated subnetwork of beta-cells [55]. Activation of GLP-1R elevates cAMP levels, which in turn promote insulin secretion via both protein kinase A (PKA-)dependent and PKA-independent mechanisms [54]. A downstream long-term effect resulting from PKA activation consists of the modification of the expression of several genes [54, 56]. Such modulation is deemed to contribute to the plasticity of the secretory response to GLP-1 and glucose. The expression of miR-375 has been shown to be controlled by the activation of the cAMP/PKA pathway [57]. Indeed, incubation of rat insulin-producing cells with the GLP-1 mimetic exendin-4 leads to a decrease in miR-375 levels in a mechanism that involves PKA [57]. Reduction of miR-375 occurs also in response to glucose but in this case in a cAMP/PKA-independent manner [57, 58]. Decrease of miR-375 in the mouse insulin-producing MIN6 cells enhances insulin secretion, whereas overexpression of this miRNA hampers the ability of the cells to secrete in response to glucose [22]. One of the targets of miR-375 that accounts for glucose-induced insulin secretion is myotrophin (Table 2) [22]. Silencing of the latter mimics the effect of miR-375 on insulin secretion [22]. Thus, the drop of miR-375 caused by glucose and exendin-4 could be beneficial for insulin secretion by increasing the expression of myotrophin [22]. Another miRNA required for insulin secretion is miR-9 (Table 2). This miRNA is upregulated during differentiation of human embryonic stem cells and during the formation of cells of both neuronal and pancreatic lineages [21, 59]. Moreover, appropriate expression of this miRNA is required for mature beta-cell tasks and probably during development. Either overexpression or silencing of miR-9 is deleterious for the secretory capacity of beta-cells [26]. In fact, manipulation of miR-9 level impinges the expression of the Onecut2 (Oc2) transcription factor, which in turn hampers the content of the secretory machinery component Slp4 [26]. As consequence of the increased level of Slp4, beta-cells insulin secretion in response to secretagogues is impaired [26].

Slp4 belongs to the Rab GTPase effector family that includes also RIM2, MyRIP/Slac2c, and Noc2 [60]. In beta-cells, these effectors are associated with Rab3a and/or Rab27a and regulate the assembly of the SNARE proteins SNAP25, Syntaxin-1, and VAMP-2, thereby controlling insulin exocytosis [60]. The expression of SNAP25, Rab3a, Rab27, and Noc2 are regulated by miR-124a in mouse insulin-producing cells [24]. Overexpression of miR-124 increases SNAP25 and Rab3a levels but reduces those of Rab27 and Noc2 [24]. miR-96 is also expressed in beta-cells and controls the expression of Slp4 and Noc2 [24]. The level of Slp4 increases and this of Noc2 decreases in cells that overexpress miR-96 [24]. The key role of miR-124a and miR-96 in the control of the level of several critical components of machinery governing insulin secretion suggests a potential participation of these miRNAs in the terminal differentiation of beta-cells.

**Figure 1:**
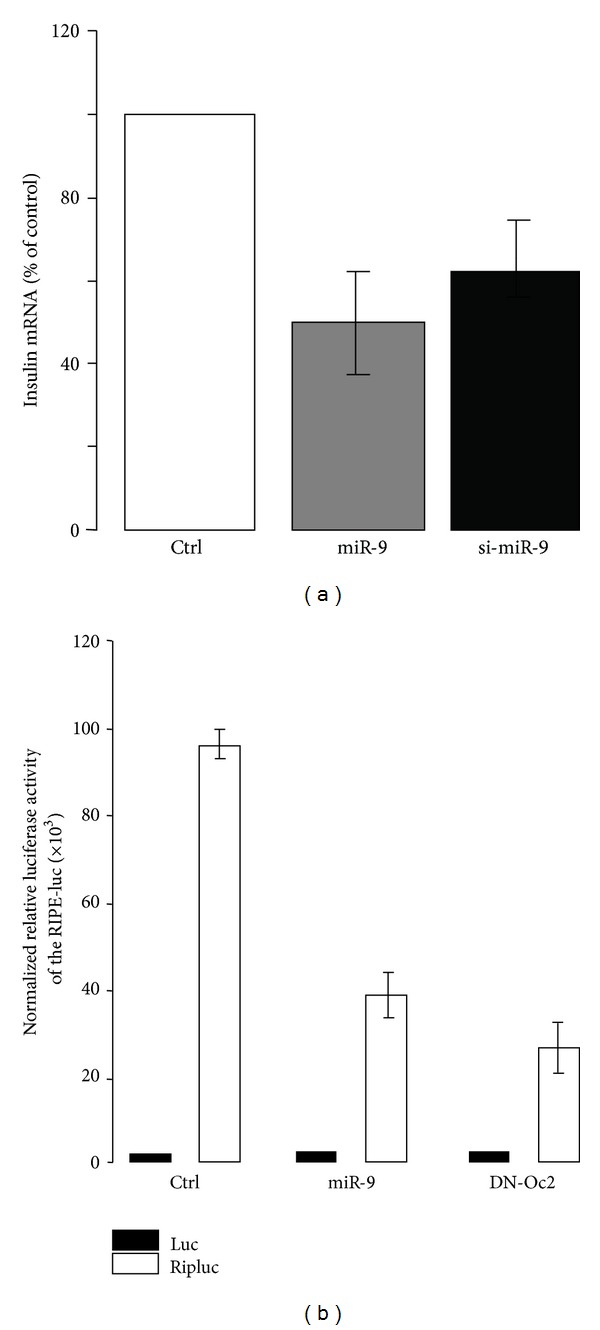
Role of miR-9 in insulin mRNA and promoter activity. (a) Effect of miR-9 on insulin mRNA. The RNA duplex containing the mature form of miR-9 [26] and a siRNA directed against miR-9 (si-miR-9) or a control oligonucleotide was transfected in MIN6 cells for 48 hrs. The expression of the preproinsulin mRNA was measured by quantitative PCR. The mRNA level was normalized against the housekeeping acidic ribosomal phosphoprotein P0 gene (*Rplp0*) and the expression level in cells transfected with the control siRNA was set to 100%. Data are the mean of ± SEM of 3 independent experiments. (b) Effects of miR-9 and dominant negative Oc2 mutant on the activity of an insulin reporter construct in MIN6 cells. MIN6 cells were transiently transfected with miR-9 RNA duplexes containing the mature form of miR-9 [26] or the dominant negative Oc2 mutant [26]. The cells were cotransfected with a luciferase reporter construct driven by a 600 bp fragment of the rat insulin promoter (Ripluc) and with pRLSV40, a construct producing a renilla luciferase activity under the control of the constitutive SV40 promoter. The firefly luciferase activity produced by Ripluc was normalized to the renilla luciferase activity to rule out differences in the transfection efficiency. The empty pGL3 basic (luc) was used as control. Each experiment was performed at least three times in triplicate.

Mature beta-cells have the exclusive task to produce insulin. At normal glucose concentrations, insulin represents approximately 1/3 of the synthesized proteins [61]. However, this ratio can reach almost 1/2 at 7 mmol/L glucose [61]. The increase of insulin production occurring within the first minutes to hours upon glucose exposure is mostly achieved through enhanced protein synthesis and mRNA stabilization. In contrast, at later time points it is mainly due to transcriptional and posttranscriptional mechanisms [62]. The long-term control of insulin mRNA levels triggered by glucose contributes to the replenishment of the hormone content and is achieved through the activation of transcriptional regulators and miRNAs. Culture of insulin-producing cells at high glucose concentration affects the expression of more than hundred miRNAs, including miR-30d which is able to increase insulin gene expression [63]. Key transcription factors involved in glucose-mediated control of insulin expression have been described in detail elsewhere [64]. These factors include v-maf avian musculoaponeurotic fibrosarcoma oncogene homolog A (MAFA), pancreatic and duodenal homeobox 1 (PDX1), and neurogenic differentiation 1 (NeuroD) [64]. MAFA abundance is under the control of miR-204. In diabetes, the beta-cell expression of this miRNA increases in response to the elevation of the cellular redox regulator thioredoxin-interacting protein. In turn miR-204 reduces the expression of insulin [65]. Regulation of insulin transcription during development involves additional transcription factors including the members of the Onecut (OC) family [66]. In view of these findings, we tested the role of Oc2 and indirectly miR-9 in the control of insulin production. We found that overexpression or silencing of miR-9 decreases insulin expression (Figure 1(a)), indicating that adequate levels of this miRNA are required for maintaining optimal insulin mRNA levels. In addition, elevated amounts of miR-9 reduce the activity of a luciferase reporter construct driven by the rat insulin promoter (Figure 1(b)), suggesting a role for miR-9 in the control of insulin gene expression. Inactivation of OC-2 using a dominant negative construct mimics the effect of miR-9 on insulin promoter (Figure 1(b)). Thus, besides regulating glucose-induced insulin secretion, miR-9 appears also to be crucial for maintaining insulin mRNA levels in a mechanism probably involving Oc2. Accumulation of insulin mRNA in response to glucose relies on nuclear translocation of PDX1. Glucose-induced nuclear import of the transcription factor is triggered by activation of the phosphatidylinositol 3-kinase (PI3K) pathway [67]. This signaling cascade results in the phosphorylation of protein kinase B by the 3-phosphoinositide-dependent kinase 1 (PDK1) [68]. Beta-cell specific knockout of PDK-1 leads to a reduction in islet cell mass and the development of overt diabetes [69]. PDK1 has been identified as a target of miR-375 [58]. Overexpression of miR-375 in rat insulin-producing INS-1E cells decreases the expression of PDK-1, leading to reduction of insulin mRNA level [58].

Numerous genes that are required for glucose-induced insulin secretion and cells survival are highly or selectively expressed in beta-cells [60]. In addition, proper control of glucose-induced insulin secretion involves the absence or the low expression of “disallowed genes” including those coding for lactate dehydrogenase A (Ldha) and monocarboxylate transporter-1 (Mct1) [70]. Overexpression of ldha in insulin secreting cells affects glucose-induced insulin secretion. Islets of individuals with diabetes display an increase in the expression of LDHA when compared to controls [70]. Beta-cells elevation of MCT1 in mice fosters pyruvate-stimulated insulin secretion, thus leading to hyperinsulinism during exercise [71]. The absence of MCT1 in beta-cells could hence prevent inappropriate insulin secretion elicited by pyruvate. A mechanism that accounts for repression of the “disallowed” MCT1 in beta-cells could involve some miRNAs. The *MCT1* mRNA is a direct target of miR-29a, miR-29b, and miR-124 [71]. From this example, it is possible that the miRNAs contribute to the silencing of “disallowed” genes in beta-cells.

## 5. miRNAs Associated with Compensatory Beta-Cell Mass Expansion in Pregnancy and Obesity

Pregnancy is the strongest physiological stimulus inducing beta-cell mass plasticity. The mass of beta-cells and their secretory activity returns to prepregnancy levels within the first 10 days following parturition in rodents [72, 73]. The levels of four miRNAs including miR-144, miR-218, miR-338-3p, and miR-451 are modified in islets from pregnant rats when compared to age-matched animals (Table 3) [74]. The expression of these miRNAs returned to resting levels after parturition [74]. *In vitro* experiments have confirmed a role for miR-338-3p and miR-451 in the control of beta-cell tasks [74]. While miR-338-3p levels are diminished, those of miR-451 are increased during pregnancy [74, 75]. Rodent and human beta-cell expansion during pregnancy is likely to occur thanks to enhanced proliferation combined with a minimal rate of apoptosis [72, 73, 76]. Overexpression of miR-451 does not increase the proliferation rate of insulin-producing cells but this miRNA protects the cells against apoptosis elicited by palmitate and cytokines [74]. Downregulation of miR-338-3p appears to be even more important in the adaptation of beta-cells during gestation. Indeed, reduction of miR-338-3p expression leads to a specific increase in proliferation of cultured insulin-producing islet cells and transplanted pseudoislets cells [74, 75]. Furthermore, as is the case for miR-451 overexpression, reduction of miR-338-3p protects the beta-cells against apoptosis evoked by diabetogenic conditions such as chronic exposure to elevated palmitate or cytokines, indicating that the decrease of miR-338-3p is pivotal for compensatory beta-cell mass expansion during pregnancy. Despite these proproliferative and antiapoptotic effects, miR-338-3p downregulation or miR-451 overexpression did not significantly impact insulin content and glucose-induced insulin secretion, indicating that upon changes in the level of these miRNAs the beta-cells retain a fully differentiated phenotype. The beta-cell mass not only increases during pregnancy but also during insulin-resistance and obesity [1]. The gain of beta-cell mass compensates for the increased insulin demand from peripheral tissues, thereby maintaining euglycemia [1]. An increase of miR-451 and a decrease of miR-338-3p analogous to those observed in islets of pregnant rats are also detected in islets of obese mice fed with high fat diet [74]. Moreover, diminution of miR-338-3p occurs in young still normoglycemic but already obese* db/d*b mice, indicating a broader role for this miRNA in physiological islet adaptation [74]. The exact mechanisms governing the expression of miR-338-3p remain to be defined. During pregnancy, the level of estradiol and incretins such as GLP-1 is elevated [77, 78] and may be responsible for beta-cell proliferation [77, 79, 80]. GLP-1 is also increased in obese individuals possibly contributing to beta-cell mass expansion [77]. Interestingly, agonists of the GPR30 estradiol receptor and of the GLP-1 receptor are able to decrease miR-338-3p levels in beta-cells via a signalling cascade involving a rise in cAMP and the activation of PKA [74].

## 6. miRNAs Associated with Beta-Cell Dysfunction under Diabetogenic Condition

The compensatory processes described above precede beta-cell decline during the development of diabetes [2, 81]. Failure in mechanisms that maintain the adaptive capacity of islet beta-cells may account for impaired beta-cell function and mass. This hypothesis has been tested by measuring the expression of miRNAs in islets of leptin receptor deficient *db/db* mice of different ages. The *db/db* mice at 6 weeks of age are obese and insulin resistant [82, 83]. However, normoglycemia is preserved and manifestation of diabetes is delayed because of increased functional beta-cell mass. Besides the decreased expression of miR-338-3p [74], the adaptive islets of these mice display variations in other miRNAs (Table 4) [84]. These include an increase in miR-132 and a decrease in miR-184, miR-203, and miR-210 [84]. Overexpression of miR-132 and inactivation of miR-184 trigger proliferation in dispersed beta-cells from rat islets [84]. In contrast, *in vitro* reduction of miR-203 and miR-210 increases rat beta-cell apoptosis [84]. The reduction of miR-210 and miR-184 is more pronounced in isolated islets from overtly diabetic *db/db* mice, suggesting that an unbalance in the level of these miRNAs can result in a switch from beta-cell adaptation to programmed cell death [84]. In addition, changes in the expression of miR-199a-3p and miR-383 appear to contribute to beta-cell failure in diabetic *db/db* mice. Indeed, upregulation of miR-199a-3p and diminution in miR-383 increase rat beta-cell apoptosis *in vitro *[84]. At the present time, the miRNAs that are associated with compensatory human islets remain to be identified. So far, one study has quantified the miRNAs level in a small group of islets of individuals with and without type 2 diabetes [85]. Only an increase in the miR-187 level is associated with beta-cell failure in diabetes [85]. This result suggests that different miRNAs account for adaptation and decline of beta-cells in human and rodents during diabetes.

Chronic elevation in circulating levels of nonesterified free fatty acids (NEFAs) is associated with obesity and is an independent predictor of T2D development [86, 87]. Numerous studies have highlighted palmitate, the most abundant NEFA in blood, as a detrimental factor promoting insulin resistance and beta-cell dysfunction. *db/db* mice display an abnormally increased blood NEFA concentration [88]. Beta-cell failure elicited by this lipid includes a decrease of insulin expression, impaired secretory capacity in response to nutrients and/or loss of beta-cell mass via apoptosis [3, 4, 89]. Elevated palmitate levels are thought to synergize with chronic hyperglycemia in promoting beta-cell failure in obesity-associated diabetes [90]. The decrease in miR-184, miR-203 and miR-383 is mimicked by chronic exposure of beta-cells to palmitate and/or glucose, suggesting a role for glucolipotoxicity in the variation of these miRNAs observed in islets of diabetic mice [90]. Additional miRNAs are changed in *db/db* mice, probably contributing to beta-cell dysfunction and death [91]. The expression of miR-34a and miR-146 is indeed augmented in islets of these mice [91]. Elevation in their levels causes dysfunction and apoptosis and mimics the harmful effects of palmitate in cultured islets [91]. Palmitate also triggers beta-cell dysfunction by an indirect mechanism that involves activation of the inflammatory process [92]. Continuous infusion of palmitate in mice evokes an increase in M1-type proinflammatory monocyte/macrophages infiltration within islets [92]. Several studies contend a role for low grade inflammation as a major issue, which links obesity to the development of diabetes [7, 89]. Interestingly, the levels of miR-34a and miR-146 are elevated by proinflammatory cytokines in isolated human islets and insulin-producing cells, indicating that the signaling cascades causing beta-cell failure elicited by palmitate and cytokines may converge and result in the activation of the same miRNAs [93]. Beside these two miRNAs, cytokines induce also the expression of miR-21 [93]. This miRNA plays a role in cell survival and can also affect glucose-induced insulin secretion by modulating the levels of components of the secretory machinery [93].

**Figure 2:**
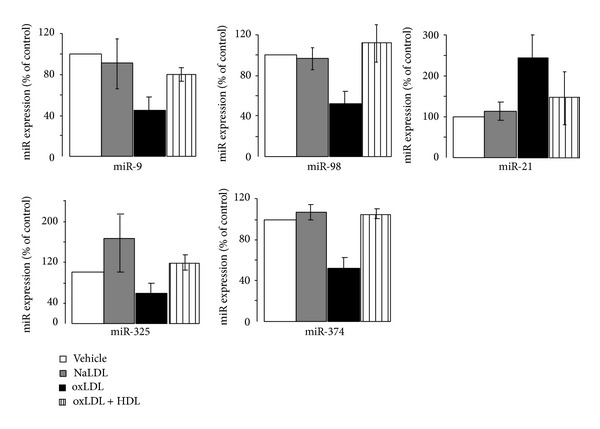
Identification of miRNAs differentially expressed in MIN6 cells cultured with human native and oxidized LDL. The expression of the indicated miRNAs was measured by quantitative RT-PCR in MIN6 cells that were cultured with vehicle, 2 mmol/L of oxidized LDL (oxLDL), and native LDL (NaLDL), plus or minus 1 mmol/L HDL-cholesterol for 72 hrs. Human plasma LDL and HDL fractions were isolated by sequential ultracentrifugation (LDL density, 1.063) as described [95]. Oxidation of LDL particles was done by incubation of 1 mg LDL protein/mL PBS with 5 *μ*mol/L CuSO4 at 37°C for 6–8 h [95]. The oxidation reaction was verified as previously described by determining the lipid peroxide content [95]. The results are expressed as fold changes and correspond to the mean ± SD.

Besides chronic hyperlipidemia and hyperglycemia, patients with diabetes display an increased ratio of oxidized LDL over native LDL [94–96]. The concentration of oxidized LDL is already elevated in prediabetic individuals [97] and increases throughout the duration of the disease [98]. The rise of oxidized LDL is thought to result, in part, from the reduced antioxidant property of HDL [94, 98–100]. Elevation of oxidized LDL apparently correlates with reduction of plasma HDL concentration, a hallmark of metabolic syndrome [101]. Importantly, infusion of recombinant HDL in patients with T2D reduces glycemia [101]. The beneficial effect of HDL relies on both improved insulin secretion and glucose uptake in muscles [101]. Several independent groups, including ours, have confirmed the protective effect of HDL against the harmful effects evoked by oxidized LDL in beta-cells [94, 95, 101, 102]. Coincubation of islets and insulin-producing cells with HDL prevents the defective insulin production and glucose-induced insulin secretion observed in the presence of 2 mM oxidized human LDL cholesterol [94, 95, 101, 102]. Moreover, cell survival is strongly improved in the presence of HDL [94, 95, 101, 102]. Although native LDL above 3.1 mM cholesterol perturbs insulin secretion and cell survival [103, 104], at 2 mM cholesterol the lipoprotein does not affect the accomplishment of the tasks and the viability of beta-cells [94, 95, 101, 102]. A global microarray profiling was done to investigate the contribution of miRNAs in the adverse effects elicited by oxidized LDL. The modified lipoprotein modified the expression of a set of 10 miRNAs (Table 5). The expression changes were further prevented by coincubation with HDL (Table 5). However, quantitative PCR analysis confirmed the variation for only 5 of them (Figure 2). The expression of miR-9 was decreased, whereas that of miR-21 was increased in insulin-secreting cells cultured with oxidized LDL particles (Figure 2). As already mentioned, upregulation of miR-21 hampers glucose-induced insulin secretion by modifying the expression of components of the secretory machinery [26, 93]. Moreover, appropriate levels of miR-9 level are required to achieve optimal insulin expression (Figure 1(b)). Thus, the changes in these two miRNAs may contribute to beta-cell dysfunction provoked by the oxidized lipoprotein. Further studies will be needed to determine whether the decrease of miR-98, miR-325, and miR-374 (Figure 2) also contributes to the loss of specific beta-cell tasks and increased death caused by oxidized LDL.

It is widely accepted that the beta-cell decline in diabetes relies on genetic factors [105]. The contribution of genetic factors varies according to the forms of diabetes. In monogenic and dominant forms of diabetes, mutations in a single gene can lead to beta-cell failure and thereby to the development of the disease [105, 106]. The maturity-onset diabetes of the young (MODY) is a familial monogenic form of early-onset type 2 diabetes, which usually develops in childhood, adolescence, or young adulthood [105]. MODY is now classified in the group of “genetic defect in beta-cell function” with a subclassification according to the gene involved [105]. The most common mutation in the gene encoding transcription factor 1 (TCF-1)/hepatocyte nuclear factor 1a (HNF1A) that causes MODY3 is a frame shift mutation in exon 4, Pro291fsinsC-HNF1A [105–107]. The mutation within the gene results in a truncated protein that plays as a dominant negative action. Overexpression of this mutant in insulin-producing cells hampers glucose-induced insulin secretion [108]. Impaired insulin secretion caused by the mutated protein is associated with an elevation in the levels of miR-103 and miR-224. Thus, genetic variation may impact the expression of miRNAs, potentially synergizing with environmental stressors in triggering islet beta-cell dysfunction in diabetes [107].

## 7. Conclusion and Perspectives

miRNAs are essential regulators of beta-cell function as evidenced by the growing number of these small RNA molecules, which play a central role in normal development, plasticity, and dysfunction of insulin-secreting cells. Besides their intracellular function, a large set of miRNAs are released in stable form in body fluids including blood and urine. Variations in the blood miRNA pool are emerging as promising biomarkers of several diseases including diabetes [109]. Indeed, circulating miRNAs including miR-103 and miR-224 have been found in the blood of patients with diabetes [108]. Transport of miRNAs within blood is achieved through different pathways involving the association with HDL particles, exosomes, and other proteins such as argonaute 2 or nucleophosmin 1 [109, 110]. Defective beta-cells can release miRNAs into bloodstream following pathophysiological conditions. Future investigations should puzzle out the physiological meaning of these circulating RNAs and determine whether the pool of miRNAs released in the blood differs according to the activation state of beta-cells. If so, monitoring these miRNAs would be insightful for monitoring whether beta-cells are in a compensatory or a failure condition. The extraordinary amount of new information provided by the discovery of the miRNAs has drawn researchers and clinical diabetologist to explore the potential involvement of another emerging class of noncoding RNAs, the long noncoding RNAs (lncRNAs) [111]. LncRNAs represent a heterogeneous population of RNA molecules longer than 200 nucleotides. The function of most of them remains unknown although several lncRNAs exert nonredundant roles in processes such as transcriptional regulation and survival [112–114]. A large number of lncRNAs is also present in human islets and some of them have their expression modified in diabetes [111]. There is no doubt that the coming years will witness the emergence of lncRNAs as additional players in the control of beta-cell function and/or in the regulation of lineage plasticity. The discovery of the regulatory potential of this emerging RNA world promises to unveil new opportunities for developing drugs capable of protecting beta-cells in the context of diabetes.

## Figures and Tables

**Table 1:** miRNAs required for beta cell specification fate and pancreas development.

miRNAs	Known functional effect	Targets	References
mir-15a, miR-15b, miR-16, and miR-195	Pancreas development, beta-cells fate and regeneration	Neurog3	[33]
miR-375	Beta- and alpha-cells expansion		[42]
miR-7a	Beta-cell proliferation	mTOR pathway components	[44, 45, 48]
miR-124a	Pancreas development	Foxa2	[33]

**Table 2:** miRNAs regulating nutrient-induced insulin secretion and insulin gene expression.

miRNAs	Known functional effect	Targets	References
miR-9	Insulin secretion	Onecut-2, Sirt1	[26]
miR-21	Insulin secretion	VAMP2, Rab3a	[93]
miR-29a, b	Insulin secretion	Mctl1	[71]
miR-30d	Insulin transcription		[63]
miR-34a	Insulin secretion	VAMP2, Rab3a	[93]
miR-96	Insulin secretion	Noc2	[24]
miR-124a	Insulin secretion	Rab27a, Noc2, MCT1	[24, 71]
miR-204	Insulin transcription	MafA	[65]
miR-375	Insulin transcription, insulin secretion	PDK1, myotrophin	[22, 58]

**Table 3:** miRNAs associated with compensatory beta-cells.

miRNAs	Cell types/models	Expression change	Known functional effect	References
miR-132	Islets of prediabetic db/db mice	Up	Beta-cells proliferation	[84]
miR-184		Down		
miR-338-3p	Islets of pregnant rats and islets of prediabetic db/db mice and obese mice fed with a high fat diet	Down	Beta-cells proliferation/antiapoptotic	[74]
	Cells cultured with estradiol or incretins			
miR-451	Islets of pregnant rats, islets of prediabetic db/db mice and obese mice fed with a high fat diet	Up	Antiapoptotic	[74]

**Table 4:** miRNAs associated with beta-cell failure.

miRNAs	Cells type/models	Expression change	Known functional effect	References
miR-21	Cells cultured with cytokines	Up	Glucose-induced insulin secretion and proapoptotic	[93]
miR-34a and miR-146a, b	Islets of diabetic db/db mice, cells cultured with cytokines or palmitate	Up	Glucose-induced insulin secretion and proapoptotic	[91, 93]
miR-184	Islets of diabetic db/db mice, cells cultured with glucolipotoxic condition	Down	Glucose-induced insulin secretion	[84, 90]
miR-187	Islets of individuals with type 2 diabetes	Up	Glucose-induced insulin secretion	[85]
miR-199a-3p	Islets of diabetic db/db mice	Up	Proapoptotic	[74]
miR-203 and miR-383	Islets of diabetic db/db mice, cells cultured with glucolipotoxic condition	Down	Proapoptotic	[90, 93]
miR-210	Islets of diabetic db/db mice	Down	Proapoptotic	[84]

**Table 5:** Global miRNA profiling of MIN6 cells cultured with human native and oxidized LDL with or without HDL. We compared by microarray analysis the expression of 350 miRNAs in MIN6 cells that were incubated with 2 mmol/L of human native (Na LDL) or oxidized LDL (oxLDL) cholesterol plus or minus 1 mmol/L of HDL for 72 hrs.

Microarray	Name	NaLDL	oxLDL	Change (log2)	Expression change	oxLDL	oxLDL + HDL	Change (log2)
NaLDL versus oxLDL	mmu-miR-9	1 622.36	739.3	−1.16	Down	2 417.08	3 407.71	0.46
	mmu-miR-21	6 073.84	15 447.81	1.37	Up	23 042.48	11 193.24	−1.01
	mmu-miR-98	7 909.01	2 817.75	−1.5	Down	12 432.59	16 691.57	0.42
	mmu-miR-192	222.59	514.66	1.02	Up	1 076.26	732.38	−0.51
	mmu-miR-325	1 018.38	462.41	−1.16	Down	1 757.68	2 642.59	0.65
	mmu-miR-342-3p	2 181.11	1 326.71	−0.73	Down	2 483.59	4 337.97	0.79
	mmu-miR-346	904.18	505.43	−0.85	Down	485.7	770.91	0.64
	mmu-miR-374	5 082.44	1 887.82	−1.43	Down	5 022.55	7 143.28	0.54
	Mmu-miR-708	366.85	712.44	0.94	up	825.11	402.52	−1.06
	mmu-miR-801	488.33	915.52	0.85	up	521.23	216.79	−1.29
